# Hot Electron‐Informed Quantum Plasmonics in Nanoparticle‐on‐Mirror Nanocavities

**DOI:** 10.1002/nap2.70173

**Published:** 2026-06-22

**Authors:** Artur Avdizhiyan, Ilya Razdolski, Anton Yu. Bykov

**Affiliations:** ^1^ Institute of Fundamental and Frontier Sciences, University of Electronic Science and Technology of China Chengdu China; ^2^ Photonics Research Centre Universiti Malaya Kuala Lumpur Malaysia; ^3^ Nanophotonics Centre, Cavendish Laboratory, Department of Physics University of Cambridge Cambridge UK

**Keywords:** Feibelman parameters, hot electrons, nanoparticle‐on‐mirror (NPoM), quantum plasmonics, surface plasmon

## Abstract

We develop a novel approach to ultrafast optical modulation of quantum‐mechanical phenomena at the interface of plasmonic metals. Focusing on efficient and versatile nanoparticle‐on‐mirror plasmonic nanocavities, we discuss indirect control of plasmonic properties through laser‐induced hot electron injection. Overcoming the limitations precluding the observations of laser‐driven mesoscopic phenomena in the time domain with state‐of‐the‐art amplified sources, our proposed experimental approach can be readily realized without irreversible optical damage and holds immense potential for the future development of ultrafast electrodynamics in nanogaps, applications in photochemistry and nanoscale control of quantum emitters. Agreeing with the results of numerical simulations, an intuitive microscopic model for the proposed time‐dependent mesoscopic electrodynamics facilitates the analysis of the temperature‐induced modulation of quantum plasmonic properties in a broad parameter space. Our work expands the realm of quantum nanophotonics onto nonequilibrium electronic systems and facilitates the development of ultrafast methods in active plasmonics.

## Graphical abstract

A computational model for ultrafast optical modulation of quantum phenomena at plasmonic metal interfaces is developed. Focusing on nanoparticle‐on‐mirror nanocavities, it describes temperature‐induced variations in electronic quantum behavior. This facilitates analysis of quantum plasmonics in non‐equilibrium systems, thereby expanding the realm of non‐equilibrium quantum nanophotonics.

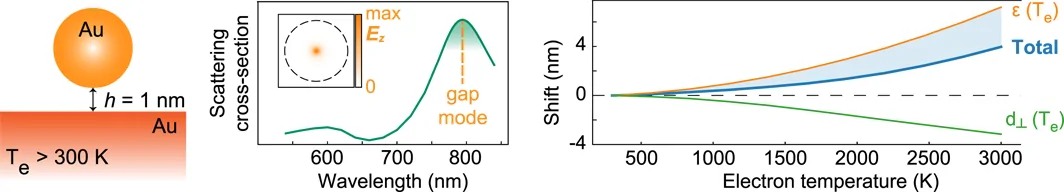

## 1 Introduction

The recent development of quantum plasmonics, an intriguing branch of nanophotonics at nano‐ and mesoscale, has led to multiple investigations of nanoobjects [1, 2, 3, 4, 5, 6]. Relevant physical processes and their spatial scales were identified, indicating different regimes where nonclassical phenomena govern the optical response of highly confined plasmonic modes [7, 8]. Lately, a physically rich landscape of quantum nanoplasmonics was complemented with the analysis of surface geometry and crystalline structures [9].

The optical response of mesoscopic quantum systems is often quantified with the Feibelman parameters [10, 11]. Providing a convenient formalism for the plasmon dispersion and a clear physical meaning, they are routinely used for the characterization of quantum‐induced optical nonlocality. Despite numerous computational methods, little is known about manipulating the quantum properties of plasmonic nanostructures through active control in the time‐domain and experimental spectroscopic evidence still remains scarce. This underscores a significant gap to the conventional local plasmonics employing the laser‐driven tuning of plasmon resonances through hot carrier excitation [12, 13, 14]. Although laser‐induced electron dynamics in metals is extensively studied, the application of hot carrier concepts to quantum phenomena at the nanoscale remains elusive due to the weakness of these perturbations to the nonlocal response and the technical difficulties in avoiding optical damage.

In this Letter, we argue that the observation of nonlocal effects in dynamics is feasible within the currently existing experimental approaches in ultrafast active plasmonics. We develop a microscopic theory relating the quantum‐driven optical nonlocality in plasmonic systems to the electron temperature and analyze the impact of temperature and Feibelman nonlocality on equal footing. Numerical simulations demonstrate the feasibility of laser‐induced modulation of the Feibelman parameters in nonequilibrium plasmonic systems and provide quantitative estimates of the hot electron‐driven optical nonlocality. We propose an experimental concept that overcomes the limitations of direct methods and enables the observation and control of mesoscopic quantum phenomena, paving the way toward active quantum plasmonics.

## 2 Theoretical Framework

We introduce the optical nonlocality through the concept of the surface‐response function [15, 16]. In real metals, the free carrier density is not localized within the sharp boundary of ionic background but is allowed to “spill out” [17], changing the surface electrostatic barrier. When subjected to high‐frequency electromagnetic radiation, this electron “spill‐out” modifies the dynamic screening at the interface and thus the optical response [18]. This behavior can be self‐consistently treated using time‐dependent density functional theory (TD‐DFT), or through the introduction of the Feibelman parameters $d_{⊥}$, $d_{\|}$ which parametrize the first moments of the induced charge and current density [11]. Multiple physical processes, including losses due to Landau damping [9, 11, 19], are encased in $\text{Im}\;d_{⊥}$ which is thus paramount for surface chemistry [20] and the development of high‐performance plasmonic devices as it reflects fundamental limits of field enhancement in plasmonic antennae [21].

Although full quantum treatment of optical nonlocality in the excited state represents a significant challenge, we herewith outline possible mechanisms that could govern the hot‐carrier‐induced effects and estimate their strength. To this end, we adapt the state‐of‐the‐art TD‐DFT approach to calculate the Feibelman $d_{⊥}$ parameter at a metal‐vacuum interface [22], which we then incorporate into rigorous numerical solution of Maxwell equations. In our TD‐DFT method, the infinite half‐space (z<0) is occupied by a prototypical plasmonic metal (gold), which is described in the local density approximation (LDA) by the bulk density corresponding to the Wigner–Seitz radius $r_{s}=3.0$ [23]. First, we solve the groundstate one‐dimensional Kohn–Sham system, self‐consistently following the standard approach for jellium systems [24]. The groundstate energies and wavefunctions are then used to construct the first‐order response function. The external potential of the incident light in the nonretarded case is taken as $\psi_{\mathrm{ext}}=-2\pi z$, and the self‐consistent solution for the induced electron density δn at a given frequency ω is as follows:

(1) $$\delta n=\int dz^{\prime }\chi \left(z,z^{\prime },\omega \right)\left[\psi_{\mathrm{ext}}\left(z^{\prime },\omega \right)+\psi_{\mathrm{ind}}\left(z^{\prime },\omega \right)+\delta V_{xc}\left(z^{\prime },\omega \right)\right],$$

where $\chi \left(z,z^{\prime },\omega \right)$ is a jellium first‐order response function in the long wavelength limit, $\psi_{\mathrm{ind}}$ is the induced Coulomb potential, and $\delta V_{xc}$ is the induced exchange‐correlation contribution prescribed in adiabatic TD‐DFT.

This framework follows the standard treatment of nonlocal surface response of simple metals extensively used in the literature [18, 24]. Importantly, it can be extended to *d*‐band metals [22, 25] by incorporating additional screening imposed by the occupied *d*‐band as a semi‐infinite slab with a dielectric function $\varepsilon_{d}\left(\omega \right)$, occupying z<0 space, so that the induced Coulomb potential satisfies:

(2) $${\left(\varepsilon_{d}\left(z,\omega \right)\frac{d\psi_{\mathrm{ind}}}{dz}\right)}^{\prime }=-4\pi \;\delta n$$

Similarly, zero frequency limit of the core electron dielectric function $\varepsilon_{d}\left(\omega =0\right)$ affects the groundstate electron density n, which we incorporate in a self‐consistent groundstate calculation through modified Poisson equation:

(3) $${\left(\varepsilon_{d}\left(z,0\right)\frac{d\psi }{dz}\right)}^{\prime }=-4\pi \;n\left(z\right)$$

This ensures consistent treatment of the electron interaction within the groundstate and response calculations. Following the solution of the response Equation (1), we derive the nonlocal response of a gold‐air interface by computing the Feibelman paramater $d_{⊥}$:

(4) $$\left.d_{⊥}=\int z\frac{d}{dz}E_{z}\left(z,\omega \right)\;dz/\int \frac{d}{dz}E_{z}\left(z,\omega \right)\;dz\right.,$$

which in practice, we compute from the induced free electron density using the dynamic force sum rule [22] (Supporting Information S1: Section 2). We note that this process can be sidestepped by utilizing the notion of nonlocal reflection coefficient [9, 22]. However, the latter requires computations at finite momenta and fitting [9] which we avoid in favor of simplicity and computational stability. In addition to the *d*‐band screening that is incorporated in Poisson Equations (2) and (3), we also introduce the finite bulk damping in our calculation by computing the response kernels at complex frequencies ω±0.5iγ. Here, γ corresponds to the Drude damping in our model which is a natural choice for a free electron system and a standard approach used in the literature [22].

To assess the role of the electron temperature $T_{e}$ in both bulk and nonlocal surface response, we supplement the above quantum‐mechanical treatment with the state‐of‐the‐art two‐band model of the temperature‐dependent dielectric function of gold [26, 27, 28]. This model independently incorporates the change in Drude damping due to electron temperature‐dependent fractional Umklapp electron scattering [29] and broadening of the interband transitions [30, 31]. The total dielectric function can be factorized as $\varepsilon \left(\omega \right)=\varepsilon_{s}\left(\omega \right)+\varepsilon_{d}\left(\omega \right)-1$ and contains an interband *d*‐band contribution accounting for the transitions around the X and L high symmetry points, higher‐order transitions prescribed by $\varepsilon_{\infty }$, and an intraband Drude term $\left(\varepsilon_{s}\right)$:

(5) $$\begin{array}{ll} \text{Im}\;\left[\varepsilon_{d}\left(\omega ,T_{e}\right)\right]= & {\left(\hbar \omega \right)}^{-2}[A_{X}J_{X}\left(\omega ,T_{e}\right)\\ & +A_{L_{4}^{+}}J_{L_{4}^{+}}\left(\omega ,T_{e}\right)+A_{L_{5+6}^{+}}J_{L_{5+6}^{+}}\left(\omega ,T_{e}\right), \end{array}$$

(6) $$\varepsilon \left(\omega \right)=\varepsilon_{\infty }+\varepsilon_{d}\left(\omega ,T_{e}\right)-\frac{\omega_{p}^{2}}{\omega^{2}+i\omega \gamma \left(\omega ,T_{e}\right)}.$$

Here, $\omega_{p}=9.02$ eV is the plasma frequency consistent with the choice of $r_{s}=3$ (see Supporting Information S1: Section 1.2), $A_{i}$ are the spectral weights of different interband transitions, and $J_{i}$ are the joint densities of states calculated by integrating the energy distribution of the joint density of states (EDJDOS) with the Fermi–Dirac occupation factors at a given $T_{e}$ [27, 28]. The damping $\gamma \left(\hbar \omega ,T_{e}\right)=\gamma_{e-e}\left(\hbar \omega ,T_{e}\right)+\gamma_{e-ph}$ contains the fractional Umklapp electron–electron and electron–phonon scattering terms. The former is given by:

(7) $$\gamma_{e-e}\left(\hbar \omega ,T_{e}\right)=\frac{\pi^{3}\beta \Delta }{12\hbar E_{F}}\left[{\left(k_{B}T_{e}\right)}^{2}+{\left(\hbar \omega /2\pi \right)}^{2}\right]$$

where Δ=0.75 and β=0.55 are dimensionless constants characterizing electron–electron scattering in noble metals [29] and $E_{F}=5.53$ eV is the Fermi energy. The band parameters are taken from Ref. [27], whereas $A_{i}$ are determined by fitting ε(ω) [26, 27] to the room temperature dielectric function of gold [32] (See Supporting Information S1: Section 2, Figure S6). To match the reported room temperature static limit for *d*‐electron screening [33], we chose $\varepsilon_{\infty }\approx 2.8$ similar to previous works [32, 34].

In total, this combined theoretical framework yields the hot electron temperature‐dependent bulk dielectric function $\varepsilon \left(\omega ,T_{e}\right)$ as well as surface functional $d_{⊥}\left[\varepsilon \left(\omega ,T_{e}\right)\right]$ that can be subsequently inserted into numerical electromagnetic simulations.

## 3 Computational Results

We calculate $d_{⊥}$ at the gold‐vacuum interface (Figure 1a) at $T_{e}=300$ K and $T_{e}=2000$ K. The negative sign of $\text{Re}\;\;d_{⊥}$ at low frequencies signifies the “spill‐in” electronic behavior typical for noble (*d*‐band) metals. Qualitatively replicating the expected behavior, the room temperature results (solid line) show a pronounced dip in $\text{Re}\;\;d_{⊥}$ around the surface plasmon frequency (2.5 eV). At elevated electron temperatures (dashed line), the dip is smeared out, reducing $\left|\text{Re}\;\;d_{⊥}\right|$ near the interband transitions. An extension of these calculations to the metal/dielectric interface is straightforward [35] and is unlikely to qualitatively change the conclusions.

**FIGURE 1:**
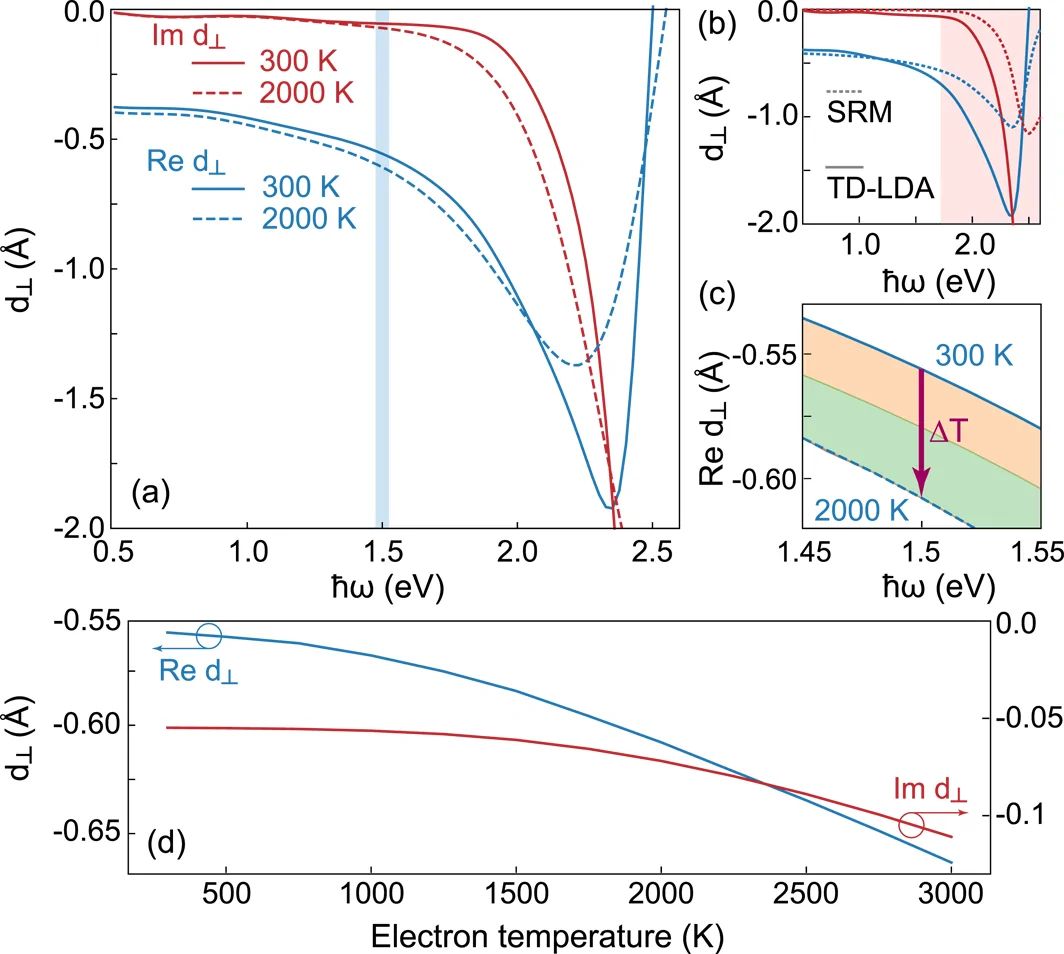
Electron temperature‐dependent nonlocal surface response. (a) Computed Feibelman parameter $d_{⊥}$ at a gold‐vacuum interface at 300 and 2000 K. (b) Comparison of TD‐LDA results (solid) with the specular reflection model (SRM, dotted). The red‐shaded area indicates the region where interband transitions dominate. (c) Zoomed‐in view of (a, blue‐shaded area) around ℏω=1.5 eV. The shaded areas in panel (c) indicate contributions from static (orange) and dynamic (green) screening to the total change of $d_{⊥}\left(\omega ,T_{e}\right)$ upon heating from 300 K (solid line) to 2000 K (dashed line). The damping contribution (red) is invisible at the current scale. (d) Dependence of $d_{⊥}$(ℏω=1.5 eV) on the electron temperature.

Below the interband transitions, where the energies of the gap plasmon modes usually reside, our results quantitatively reproduce those obtained from the semiclassical specular reflection model (SRM, Figure 1b), which have been shown to match the results of the quantum‐mechanical calculations with ad hoc carefully constructed atomic layer potentials [9]. Above the interband transition threshold the calculations diverge since our free‐electron model only accounts for the screening from the *d*‐band. Although still remaining a simple one‐dimensional model, this work represents the first quantitative self‐consistent TD‐DFT calculation of optical nonlocality at the gold surface in the experimentally relevant domain.

When the electron temperature increases, the damping modulation and broadening of the interband transitions modifies the contribution of bound electrons to ε [26, 36]. At low frequencies, ε changes due to the Kramers–Kronig compliance and fractional Umklapp electron–electron scattering [33, 36, 37]. The increase in the real part introduces corrections in both static and dynamic screening within our model, thus emphasizing the “spill‐in” behavior. The surge of the damping rate (and correspondingly, Imε) modifies the resonance broadening at 2.5 eV. Since the shift of the surface plasmon to 2.5 eV from $0.8\;\omega_{p}\approx \;7.2$ eV is determined by *d*‐band screening, the modification of $d_{⊥}$ due to the electron heating is most pronounced in this region. However, a significant tail extends toward lower frequencies, with ≈10% variations observed around the typical gap plasmon mode energy (1.5 eV). The developed microscopic theory thus predicts that in the “surface plasmon” frequency range (∼1−2 eV) $\text{Re}\;\;d_{⊥}$ in gold becomes more negative with temperature, indicating that hot electrons exhibit stronger spill‐in.

Experimental observation of these phenomena is often complicated by their smallness. Generally, nonlocal corrections in plasmonic nanoobjects of size R are considered small together with the $d_{⊥}/R$ ratio [38, 39]. On the contrary, in geometries with narrow gaps (width h∼1 nm), much stronger $\left(∼d_{⊥}/h\right)$ nonlocality can be expected. In a nanoparticle‐on‐mirror (NPoM) class of plasmonic systems, a nanocavity is formed by a gold particle placed over the surface (mirror) of a thin (usually 50–100 nm) gold film. Such nanocavities can be readily produced via chemical means by drop‐casting nanoparticle solutions on gold surfaces coated with self‐assembled molecular monolayers [40]. Using alkane‐thiols [40] or cucurubiturils [41], reproducible nanogaps with thicknesses down to 1 nm can be fabricated. Larger gaps can be routinely produced with longer‐chain molecules [42] or atomic‐layer‐deposited oxide films [16], whereas even thinner spacers (∼0.4 nm) are also feasible with the use of 2D materials [43]. Numerical calculations indeed demonstrate a strong impact of nonlocality in narrow‐gap plasmonic NPoMs [44]. However, low damage threshold of plasmonic nanocavities and strong (up to $10^{3}$) field localization and enhancement in the gap [45] makes active modulation of NPoM optical properties with ultrafast laser pulses extremely challenging. This is further exacerbated by the somewhat malleable nature of gold producing flares [46] and picocavities [47] in strong electric fields. We will address these experimental difficulties in the concluding section of this paper.

## 4 Numerical Simulations

An inherent connection between the electronic temperature and nonlocality of the optical properties at a metal‐dielectric interface enables quantification of the resulting variations of the optical response. Due to the complexity of joining numerical electrodynamics with the quantum‐mechanical calculations in the excited state, we first consider $d_{⊥}$ as an independent control parameter. Its dynamic nature is qualitatively justified with the microscopic model above. The above calculations represent properties of a gold‐vacuum interface and thus can be directly employed for numerical simulations of an arbitrary plasmonic system.

Using the finite element method (FEM) implemented in COMSOL Multiphysics, we modeled the optical response of gold nanosphere‐on‐mirror (NPoM) structures, incorporating mesoscopic boundary conditions via weak‐form integrals within the scattering formulation of Maxwell's equations, following the approach in Ref. [16] (see also Supporting Information S1: Section S2). The NPoM configuration (Figure 2a) consists of a gold nanosphere (radius R=50 nm) separated from a gold mirror layer by a nanoscale gap (thickness h=1–5 nm) filled with a dielectric of permittivity $\varepsilon_{g}=2$. The 50 nm thick gold mirror rests on a 200 nm insulating layer (ε=2.25). A three‐dimensional computational domain (rectangular cuboid) with lateral dimensions of 500 nm is used, enclosed by a 150‐nm‐thick perfectly matched layer (PML) to ensure ideal absorption outside the main simulation region. Illumination is provided by *p*‐polarized radiation at an incidence angle $\theta_{\text{inc}}=52{}^{\circ}$. Each gold–air interface is characterized by the wavelength‐ and temperature‐dependent Feibelman parameter $d_{⊥}\left(\lambda ,T_{e}\right)$, whereas $d_{\|}=0$ due to charge neutrality. In nonlocal calculations, $d_{⊥}$ is applied simultaneously to both the mirror and the nanoparticle. The nanoparticle temperature is kept at 300 K, and its optical permittivity is taken from Ref. [32]. Numerical tests at room temperature show excellent continuity in the $d_{⊥}\to 0$ limit, with perfect agreement to local‐response calculations (Supporting Information S1: Figure S1). The finite‐element mesh contains approximately $8\times 10^{5}$ nonuniformly distributed elements, with a minimum element size of 0.06 nm. The model is deployed on a high‐performance computing node with 512 GB of system memory, enabling in‐memory handling of fine discretizations and complex geometries as well as efficient assembly and solution of large sparse linear systems. COMSOL routines extract key optical observables, including scattering and absorption cross‐sections and near‐field electric field enhancements. To evaluate the impact of the temperature dependence of Feibelman nonlocality in advanced plasmonic systems, we systematically calculated dark‐field scattering spectra of these NPoMs in a wide parameter space.

**FIGURE 2:**
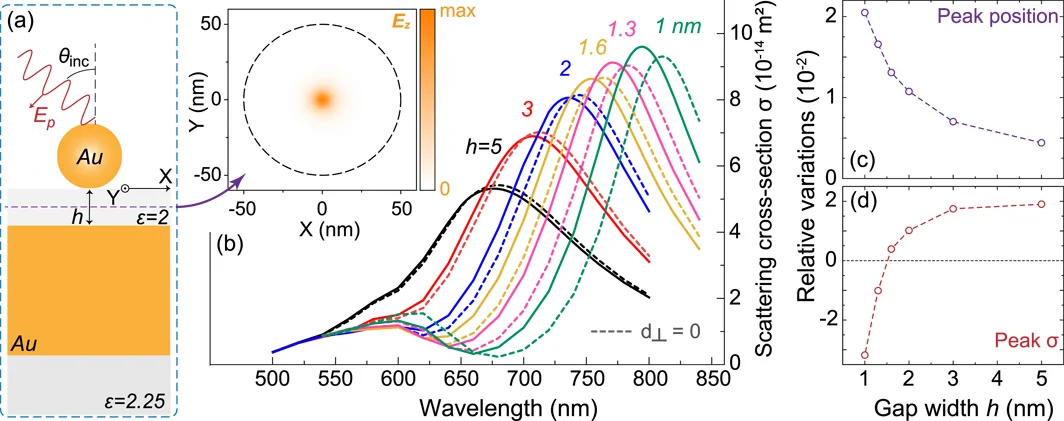
Role of nonlocality and gap size in gold NPoM: (a) Schematics of the COMSOL computational model featuring a NPoM system with the gap filled by a lossless dielectric with ε=2. (b) Scattering cross‐sections of NPoMs with varied gap width in local theory (dashed lines) and nonlocal theory with $d_{⊥}$ taken from Ref. [9] (solid lines). The inset (top‐left) shows the calculated in‐plane distribution of the normal component of the electric field $\left|E_{z}\right|$ in the middle of the gap indicated by the dashed line in panel (a) for h=1 nm, $d_{⊥}=0$ at λ=812 nm, proving the gap nature of the plasmon mode. The circular dashed line indicates the nanoparticle size; (c, d) Relative variations in peak position Δλ/λ (c) and scattering amplitude at the maximum Δσ/σ (d) between the local and nonlocal theories.

Figure 2b shows calculated scattering spectra of NPoMs where, upon reducing the gap width h from 5 to 1 nm, the resonance redshifts [40]. This trend is reproduced in both nonlocal ($d_{⊥}\neq 0$, solid lines) and local ($d_{⊥}=0$, dashed lines) calculations. The impact of nonlocality increases toward smaller gaps (Figure 2c): NPoMs with h=1 nm demonstrate the strongest blueshift when $d_{⊥}\neq 0$ is introduced and thus are the most promising for active modulation. This blueshift of the plasmon resonance in nonlocal systems is consistent with the spill‐in electron behavior characteristic for noble metals below the onset of interband transitions [16, 48]. Although NPoMs with even smaller gaps might exhibit larger effects, in practice the gap thickness uniformity becomes a challenge. Furthermore, since at h≲0.5−1 nm electron tunneling becomes important [7, 49], we focus on the 1 nm gap geometry.

To set up a reference point for the nonlocality, we employ $d_{⊥0}\approx -0.48$ Å obtained [9] for gold at λ=790 nm, that is, in the vicinity of the gap plasmon mode at h=1 nm. The impact of the electronic temperature in the mirror $T_{e}$ and nonlocality $d_{⊥}$ is seen in Figure 3a,b. The resonant wavelength exhibits much stronger sensitivity to the Feibelman nonlocality than the linewidth (cf. Supporting Information S1: Figure S6). Naturally, this is the result of our assumption $\text{Im}\;d_{⊥}=0$ justified by the microscopic description below the onset of interband transitions. Both temperature rise and an increase of the electron spill‐out redshift the gap mode. Please note that because $d_{⊥0}<0$, the horizontal axis in Figure 3d points in the direction of further decrease of $d_{⊥}$, that is stronger spill‐in, thus relating spill‐in behavior to the resonance blueshift, in agreement with the conventional quantum plasmonic framework [16, 48]. This dependence, characteristic for quantum plasmonic systems, is usually expanded into a series and truncated at the linear term, treating $kd_{⊥}$ as a small parameter [4, 50, 51]. The extracted peak positions (Figure 3d) indeed show a dominant linear contribution with a temperature‐independent slope of $\partial \lambda /\partial d_{⊥}\approx 290$. However, owing to the strong variations of $d_{⊥}$ in our simulations, a next term in the expansion, that is a second order nonlocal correction $\partial^{2}\lambda /\partial d_{⊥}^{2}\approx 1800$ ${\text{nm}}^{-1}$ is also discernible from our results (Supporting Information S1: Figure S4). To our knowledge, this is the first identification of the second‐order Feibelman‐like contribution, indicating exceptional susceptibility of the gap plasmons in nonequilibrium quantum plasmonics.

**FIGURE 3:**
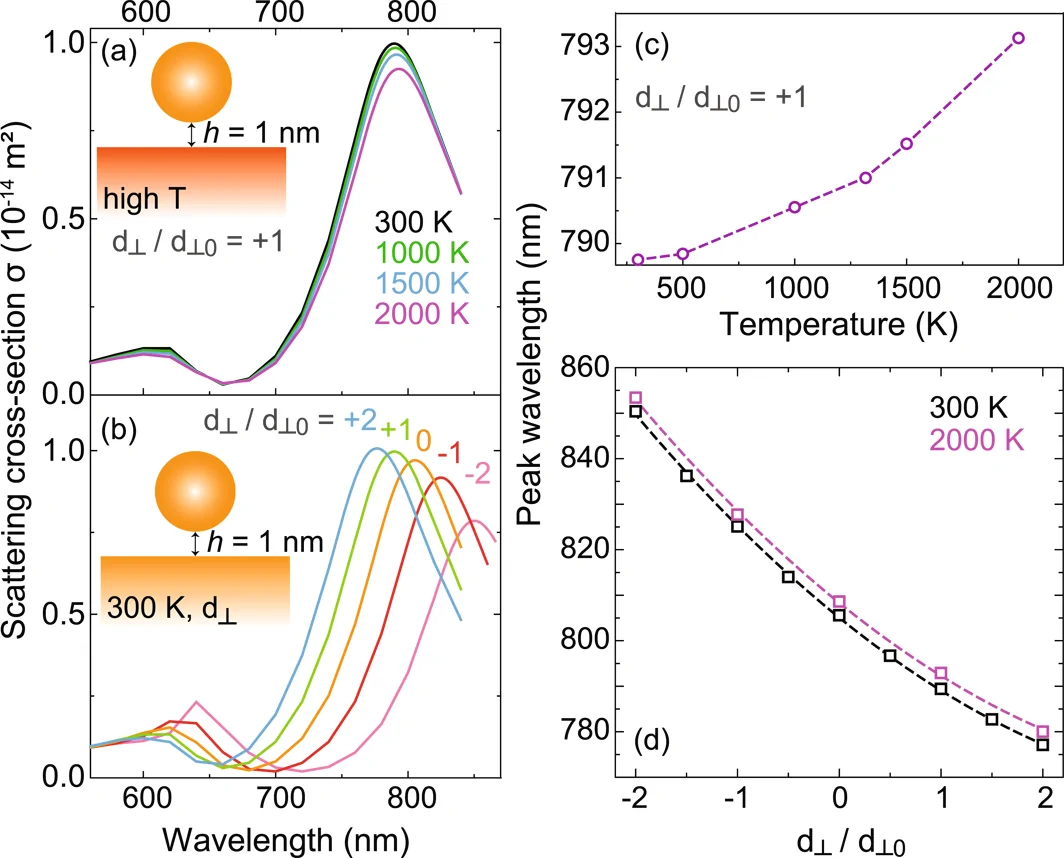
Interplay of nonlocality and substrate heating in Au NPoMs with the gap width h=1 nm. (a, b) Scattering cross‐section spectra of NPoMs with: (a) varied mirror temperatures and fixed $d_{⊥}=d_{⊥0}$, and (b) cold mirror at 300 K and varied $d_{⊥}$. (c, d) Extracted scattering peak positions at various mirror temperatures and $d_{⊥}/d_{⊥0}=1$ (c), and nonlocality $d_{⊥}$ in the mirror at $T_{e}=300$ K and 2000 K (d).

## 5 Analytic Quantum‐Corrected Circuit Model

It is instructive to incorporate NPoM nonlocal corrections into a simple analytic theory capable of replicating the numerical results qualitatively. Recall that the gap mode is the ultimate case for the hybridized surface plasmons at the two adjacent interfaces [45]. Resonant plasmonic nanocavities can be modeled as effective electric circuits at optical frequencies [52], where NPoM is replaced with a nanoparticle dimer for convenience. The use of a dimer antenna representing a particle and its image is a standard approach that has been successfully applied to NPoM systems in the past [53]. This coupled oscillators model exhibits two resonances corresponding to the LSPR of the individual nanoparticles and the gap mode, respectively. The latter is enabled by the capacitive coupling $C_{g}$ between the LC‐circuits on the particles.

Adapting the model to capture the nonlocal response, we need to account for both $d_{⊥}/R$ and $d_{⊥}/h$ corrections. The former is realized with a surface capacitance $C_{\text{surf}}$ (Supporting Information S1: Section 1, Figure 4a). Employing the Drude model with $\varepsilon_{\infty }$ and $\omega_{p}$, the resonance wavelength of the gap plasmon is as follows:

(8) $$\lambda_{0}=\lambda_{p}\sqrt{\varepsilon_{\infty }+2\varepsilon_{h}\left(1+2\eta \right)\frac{1+d_{⊥}/R}{1-2d_{⊥}/R}},$$

where $\eta =C_{g}/C_{s}$ is the ratio of the gap and sphere capacitances, $\varepsilon_{h}=1$ is the host medium dielectric function and $\lambda_{p}=2\pi c/\omega_{p}$.

**FIGURE 4:**
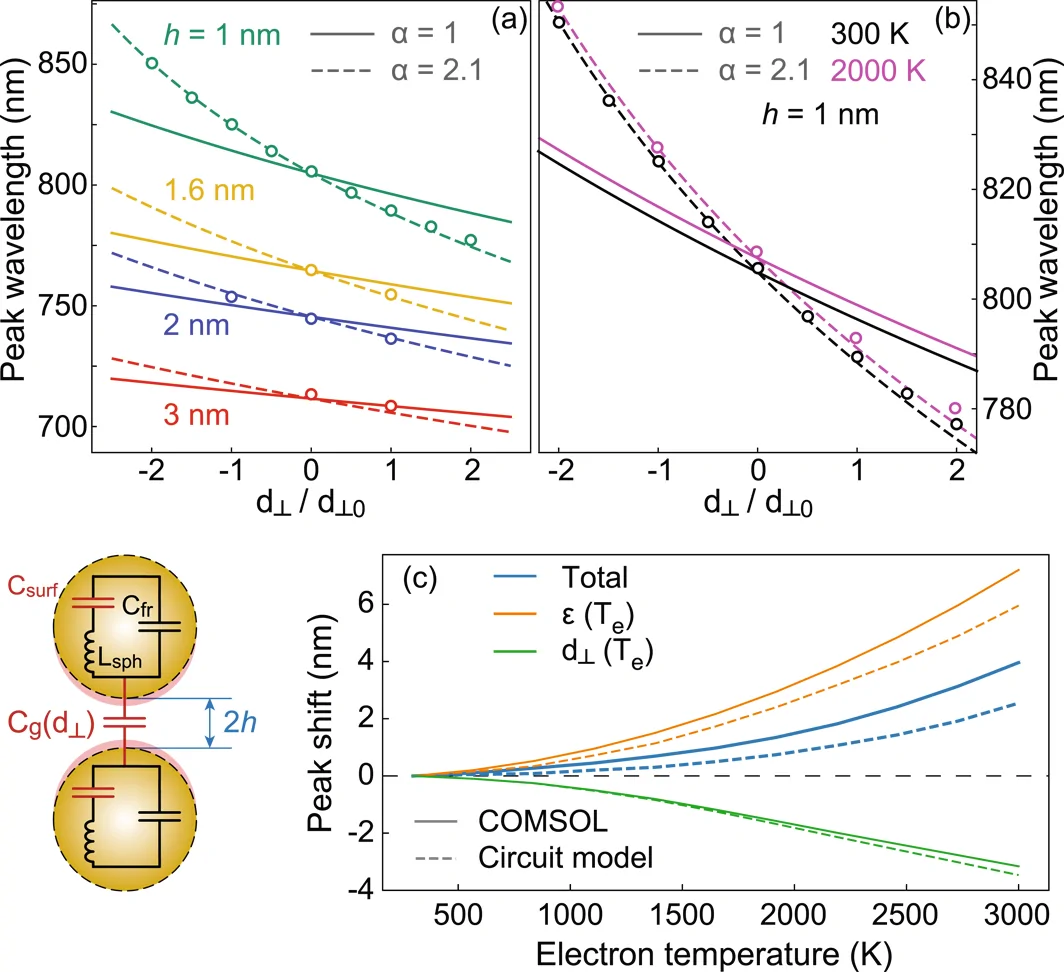
Variations of the calculated resonant wavelengths with the Feibelman parameter $d_{⊥}$ for different gap widths (a) and temperatures of the gold mirror (b) with the analytic circuit model. The dashed and solid lines represent the expected scaling of the gap capacitance with $d_{⊥}$ with and without the correction factor α=2.1, respectively. The inset in the bottom‐left corner shows a schematic circuit of a nanoparticle dimer with nonlocality. The red‐colored $C_{g}$ is modified through the Feibelman nonlocality $d_{⊥}$ which can be described by the effective gap width renormalization (Equation 9, red‐shaded spheres). (c) Calculated resonance shifts with temperature: the impact of bulk ε (orange) and Feibelman nonlocality $d_{⊥}$ (green) with temperature, and the total shift (blue). The solid and dashed lines indicate the results of numerical simulations and the circuit model, respectively.

In conventional NPoM geometries, R≫h, thus emphasizing the $∼d_{⊥}/h$ corrections. In Equation (8), they are responsible for the modification of the effective gap width h thereby adjusting the ratio of capacitances η. The latter in the NPoM geometry can be calculated as follows [53, 54] (see also Supporting Information S1: Section 1.3):

(9) $$\eta =\frac{n_{g}^{\chi }}{\varepsilon_{h}}\ln\left(1+\frac{R}{2\left(h-2\alpha d_{⊥}\right)}\theta_{\max}^{2}\right).$$

A phenomenological parameter α serves to compensate the model's underestimation of the role of $d_{⊥}$ due to local assumptions underlying the derivation of Equation (9). Because the physical meaning of $d_{⊥}$ shows the displacement of the induced electronic density from the (sharp) interfaces, this signifies a tempting approach to introduce an effective renormalization of the gap width due to the spill‐in (or ‐out) of the electron density. Within the model, $\varepsilon_{\infty }$ and χ are used to match the resonant frequencies of nanocavities for $d_{⊥}=0$. We estimated $\varepsilon_{\infty }$ from the LSPR scaling in water (Supporting Information S1: Figure S1), incorporating subtle retardation effects [53]. The role of $T_{e}$ can be accounted for by calculating the $\text{Re}\;\varepsilon_{Au}$ variations using the microscopic theory (Equations (5), (6), (7)) whereas we corrected $\varepsilon_{\infty }$ by Δε=Reε(2000K)−Reε(300K)≈0.22. The base theory (Figure 4a,b, α=1) severely underestimates the role of nonlocality. Yet, an excellent agreement of the analytic theory with the numerically computed resonance wavelengths at both $T_{e}$ and various gap widths is obtained by setting α=2.1, even though the dimer‐based circuit model does not directly incorporate the heated mirror‐induced asymmetry. Moreover, the model is capable of replicating the nonlocal behavior in the wide $h-d_{⊥}$ parameter space (Figure 4a). Supporting our numerical treatment, this result introduces an intuitive analytic way to model nonlocal plasmonic nanocavities out of thermal equilibrium.

The need for a correcting coefficient α can also be inferred from comparing the sensitivity of the gap plasmon wavelength $\lambda_{0}$ to the variations of h and $d_{⊥}$ in numerical simulations. In the vicinity of h=1 nm (Figure 2), we find $\partial \lambda_{0}/\partial h\approx -118$. Recalling the variations of the resonance position with $d_{⊥}$ from Figure 3d, for the relative sensitivity we obtain $\xi \equiv \left(\partial \lambda_{0}/\partial h\right)/\left(\partial \lambda_{0}/\partial d_{⊥}\right)\approx -0.41$. Although at smaller gaps $\left|\partial \lambda_{0}/\partial h\right|$ surges, ξ>−1 still holds in the practical range of h, indicating a more complicated gap renormalization in the presence of nonlocal plasmonic response.

We can now illustrate the heating‐induced shifts of the gap plasmon resonances as predicted by both analytic model and numerical simulations. In both cases, we calculate contributions from the temperature‐induced variations of the bulk ε and nonlocality $d_{⊥}$ as well as the total shifts (Figure 4c). Although the analytic circuit model suffers from multiple assumptions and simplistic parametrization, it yields a good qualitative agreement with the numerical results.

Both approaches show that the $d_{⊥}\left(T_{e}\right)$ dependence significantly reduces the resonance shift at elevated temperatures (∼50%), enabled by the opposite signs of the $\varepsilon \left(T_{e}\right)$ and $d_{⊥}\left(T_{e}\right)$ contributions. Whereas the former dependence is well established, the latter provides important insights into the nonequilibrium nonlocality of the optical response of nanoplasmonic systems with noble metals. The calculated reduction of the temperature‐induced resonance shifts is directly observable in the experiment, for example with ultrafast laser‐induced heating of the plasmonic system. In the calculations, modifications of the spill‐out of high‐$T_{e}$ electrons are considered statically, assuming a quasi‐instantaneous response of the electron density to the temperature variations. To monitor temporal evolution of the optical nonlocality, this framework can be complemented by established models for the electron dynamics, for example two‐temperature model [55] with a hot‐electron expansion [36, 56, 57] featuring a laser pulse as an energy source. From there, time traces for $T_{e}$ and the energy of the electron subsystem can be obtained, thus enabling modeling of the electron spill‐out dynamics $d_{⊥}\left(t\right)$ in nonequilibrium conditions. The ε‐driven contribution (orange) can be estimated in other plasmonic systems where the role of nonlocality is suppressed. This work thus provides a comprehensive framework for the active ultrafast nonlocal plasmonics.

## 6 Toward Experimental Observations

Lastly, we briefly discuss relevant experimental concepts which can be employed for the observation of the calculated effects. Conventional methods for creating hot electrons in a metal with an ultrashort laser pulse are unsuitable due to the low damage threshold of the nanoparticle. Indeed, excitation power density sufficient to heat up electrons in the Au mirror up to few thousand Kelvin is likely to inflict irreversible damage to the NPoM [45].

The problem of direct optical heating of the fragile nanoparticle can be avoided in a bilayer configuration (Figure 5) based on the NPoM geometry with a thick (50–100 nm) gold film epitaxially grown on top of a thin (5–10 nm) layer of iron [58, 59, 60, 61]. The Fe emitter is irradiated with an ultrashort laser pulse through a transparent substrate (not shown). The high transmittance of the high‐quality Fe/Au interface for laser‐excited electrons [62] results in their efficient injection from Fe into Au. Laser‐excited Fe/Au bilayers generate sub‐100 fs bursts of ballistic electron propagating though Au at the Fermi velocity $v_{F}∼1$ nm/fs as demonstrated in multiple studies of ultrafast spin‐polarized transport over 50–100 nm [58, 60, 62, 63, 64]. Due to the large ballistic length (∼100 nm) [59], the injected electrons traverse the gold layer, enabling transient nonequilibrium electronic configurations in the proposed NPoM geometry where electronic asymmetry between the mirror and the particle is introduced. The highly energetic electrons reaching the front surface of the gold mirror modulate $\varepsilon_{\text{Au}}$ [59] and, potentially, the spill‐out effects at the interface [61] while leaving the nanoparticle unperturbed. The dynamics of electron spill‐out and other relevant quantum phenomena can be monitored through the transient dark‐field scattering of the NPoM plasmonic nanocavities. As an important practical advantage, our approach enables strong perturbations of the electronic density without an optical damage to NPoM since the probe light can be made relatively weak.

**FIGURE 5:**
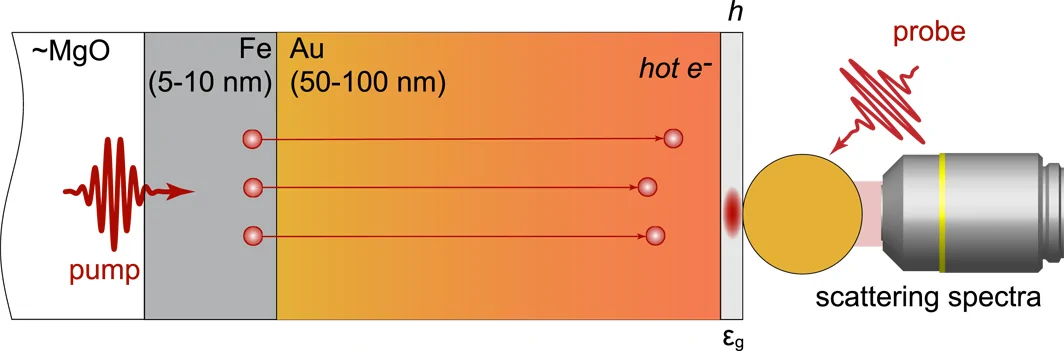
Proposed experimental configuration. An ultrashort pump pulse is absorbed in the Fe layer, inducing hot electron injection from Fe into Au and their subsequent ballistic transport across the Au layer [58]. Collected with a high‐NA objective and a monochromator, the delayed probe pulse monitors transient variations of the plasmonic (glowing red hotspot) spectral response of an NPoM nanocavity. The latter is formed by a spherical Au nanoparticle resting on a dielectric layer with a thickness h and dielectric function $\varepsilon_{g}$.

Importantly, the proposed hot electron‐driven mechanism might not be the only one responsible for the ultrafast modulation of the optical nonlocality in these experimental conditions. Variations in electron density and changes in the dielectric constant as a result of molecular motion in the gap, chemical transformations, and surface charging can all contribute to control both classical and quantum plasmonic phenomena at the ultrafast timescale. Moreover, the case of elevated electron temperature considered here thermodynamically refers to the situation where the excited electronic subsystem has already reached the Fermi–Dirac equilibrium at $T_{e}$ but thermalization with the cold lattice has not started yet. In typical plasmonic metals, this state can be expected at a few hundred fs delay after the exposure to a short (≲50 fs) excitation pulse. We thus cannot rule out stronger modulation of the plasmon resonance frequency at shorter time delays when the electronic state is nonthermal.

## 7 Conclusions

In conclusion, we calculated thermal variations of the Feibelman nonlocality in gold and proposed an experimental approach to actively control nonclassical plasmonic phenomena in nanogaps with ultrafast laser‐induced hot electron bursts. Our analytical and numerical results indicate sizeable shifts of a fundamental gap plasmon mode in NPoM nanocavities that could be monitored with state‐of‐the‐art ultrafast spectroscopy. Minimizing the optical damage to the nanocavities, this approach facilitates the development of active quantum plasmonics and other fundamental studies of high‐energy and nonequilibrium phenomena. It might prove vital in studying coherent molecular motion in nanogaps [65], picocavities [47], and dynamic strong coupling [66] as well as probing hot carrier dynamics with atomic resolution [67]. The asymmetric nature of the predicted modification is interesting for the fundamental studies of nanocavities with broken symmetry, that is made of a combination of “spill‐in” and “spill‐out” metals [16] such as recently developed robust magnesium nanoparticles [68] on gold mirrors. Furthermore, a feasible integration of the proposed geometry with 2D materials opens the door to the fascinating perspectives of dynamic control of electron tunneling [7]. Taking advantage of the single‐molecule sensitivity of NPoM plasmonic cavities, our proposal holds promise for advance studies of hot carrier‐driven chemistry and ultrafast catalysis in nanogaps.

## Author Contributions

**Artur Avdizhiyan:** investigation, writing – review and editing, methodology, software, formal analysis, resources. **Ilya Razdolski:** investigation, funding acquisition, writing – original draft, conceptualization, methodology, supervision, project administration, resources, writing – review and editing, visualization, validation, formal analysis, data curation. **Anton Yu. Bykov:** writing – original draft, investigation, conceptualization, visualization, writing – review and editing, validation, methodology, formal analysis, software, supervision, data curation.

## Supporting information


Supporting Information S1

## Data Availability

The data that support the findings of this study are available from the corresponding authors upon reasonable request.
