# Supplementary material for: Hot Electron‐Informed Quantum Plasmonics in Nanoparticle‐on‐Mirror Nanocavities
Source: Nanophotonics. 2026 Jun 22;15(12):e70173. doi: 10.1002/nap2.70173 (PMC13642080; doi:10.1002/nap2.70173)
Supplement: Supplementary file 1 [file NAP2-15-e70173-s001.pdf]

Supporting Information

Non-equilibrium quantum plasmonics in nanoparticle-on-mirror nanocavities

Artur Avdizhiyan,[†] Ilya Razdolski,[*,‡] and Anton Yu. Bykov[*,¶]

†*Institute of Fundamental and Frontier Sciences, University of Electronic Science and Technology of China, Chengdu 610054, P. R. China*

‡*Photonics Research Centre, Universiti Malaya, 50603 Kuala Lumpur, Malaysia*

¶*Nanophotonics Centre, Cavendish Laboratory, Department of Physics, University of Cambridge, CB3 0US, UK*

E-mail: ilya@um.edu.my; ab3228@cam.ac.uk

S1 Theory

S1.1 Nonlocal circuit model for NPoM

To implement a simple analytic model that would qualitatively predict the nonlocal response of a NPoM plasmonic cavity, we adapt the technique pioneered in[1] treating nanoparticles as nanoscale curcuit elements at optical frequencies.

S1.1.1 Isolated plasmonic nanoparticle

The starting point is the derivation of the electric field of an individual metallic sphere described by the dielectric function $\varepsilon_m(\omega)$, in the dielectric environment with ε_d in a uniform

external electric field. For this we recall the general solution of a Laplace equation in spherical coordinate system:

$$\phi(r,\theta) = \sum_{l=0}^{\infty} \left(A_l r^l + \frac{B_l}{r^{l+1}} \right) P_l(\cos\theta), \tag{S1}$$

where P_l are Legendre polynomials. To retain the simplicity and since we are only interested in the main dipole feature of the scattering spectrum, we restrict ourselves to the simplest quasistatic approximation and, assuming the external field $\mathbf{E}_0||\mathbf{z}$, seek the electrostatic potential in the form:

$$\begin{aligned} \phi_{in}(r,\theta) &= Ar\cos\theta, \\ \phi_{out}(r,\theta) &= E_0 r\cos\theta + \frac{B}{r^2}\cos\theta, \end{aligned} \tag{S2}$$

Applying the standard boundary conditions – continuity of the potential and radial displacement D_r at $r = R$ (radius of the nanoparticle) – yields the textbook solution for this problem. Extension of this approach to include nonlocal corrections within the Feibelman framework is straightforward, and involves replacing continuity of the potential with the new boundary condition:

$$E_\theta^m - E_\theta^d = -d_\perp \nabla_\parallel \left(E_r^m - E_r^d \right) \tag{S3}$$

This condition, after straightforward algebra, yields the following result:

$$\begin{aligned} \mathbf{E}_{in} &= \frac{3\varepsilon_h}{\varepsilon_m + 2\varepsilon_h - 2d_\perp \left(\varepsilon_m - \varepsilon_h\right)/R} \cdot \mathbf{z}, \\ \mathbf{E}_{out} &= E_0 \cdot \mathbf{z} + \frac{3\mathbf{r}(\mathbf{p}\cdot\mathbf{r}) - \mathbf{p}}{\varepsilon_h R^3}, \end{aligned} \tag{S4}$$

where ε_m and ε_h are the metal and host medium dielectric functions, respectively, and the

nonlocal dipole moment of the nanoparticle is given by:

$$(\mathbf{p}\cdot\mathbf{z}) = \varepsilon_h R^3 \frac{\varepsilon_m - \varepsilon_h + d_\perp(\varepsilon_m - \varepsilon_h)/R}{\varepsilon_m + 2\varepsilon_h - 2d_\perp\left(\varepsilon_m - \varepsilon_h\right)/R} \tag{S5}$$

It is clear that these expressions retain the overall structure of the local solution, including a representation of the field outside the particle as a sum of the external and dipole fields, and converge to the known answer in the absence of nonlocality. We would also like to point out that nonlocal polarisabilities could be similarly derived for an arbitrary order in the multipolar expansion.[2]

With the expression of the fields known, we can adapt the circuit model from Ref.[1] to our problem. Identically, the continuity of D_r allows to formulate the "effective" Kirchhoff current law for the system:

$$-i\pi\omega(\varepsilon_h - \varepsilon_m)R^2 E_0 = -2i\pi\omega\varepsilon_h R^2 E_0\left(1 + \frac{d_\perp}{R}\right)\Gamma - i\pi\omega\varepsilon_m R^2 E_0\left(1 - \frac{2d_\perp}{R}\right)\Gamma, \tag{S6}$$

with $\Gamma = (\varepsilon_m - \varepsilon_d)/(\varepsilon_m + 2\varepsilon_h - 2d_\perp(\varepsilon_m - \varepsilon_h)/R)$. As in Ref.,[1] we interpret the first and second terms on the right as fringe and sphere current respectively, however due to boundary condition (S3) introducing a discontinuity of the potential we can no longer satisfy the Kirchhoff voltage law with just two circuit elements. Instead, we introduce a new element with the impedance Z_{surf}, which is connected in series with the Z_{sphere}, and serves to incorporate a potential jump due to the additional dipole layer at the interface. After computing the average potential difference between the upper and lower halves of the sphere we can express the impedances of all three circuit elements as follows:

$$Z_{\text{sphere}} = (-i\pi\omega\varepsilon_m R)^{-1}, \quad Z_{\text{fringe}} = (-2i\pi\omega\varepsilon_h R)^{-1}, \quad Z_{\text{surf}} = \frac{3d_\perp}{-i\pi\omega\varepsilon_h R^2}\left(1 - \frac{2d_\perp}{R}\right)^{-1} \tag{S7}$$

This result allows a clear interpretation of the role of nonlocality. For the noble metals at low frequencies, where $d_\perp$ is real and negative, this circuit element represents an additional capacitance (or negative inductance) connected in series to the classical inductance of the nanoparticle. It therefore serves to increase the resonance frequency of the corresponding LC circuit, as reported elsewhere.[3]

S1.1.2 Coupled circuit model for the NPoM

The developed non-local circuit model allows us to explore coupled plasmonic geometries. As in the previous works[4] we treat the nanoparticle-over-mirror as a dimer produced by coupling of the nanoparticle plasmon with its image charges. Following the smae approach, we model this coupling by introducing an LRC circuit where L_g, C_g and R_g represent the effective inductance, capacitance and resistance of the gap, respectively. The total impedance of the system Z_{total} is then given by:

$$Z_{\text{total}} = \frac{2\,(1 + d_\perp/R)}{-i\pi\omega R\,(\varepsilon_m + 2\varepsilon_h - 2d_\perp(\varepsilon_m - \varepsilon_h)/R)} + \frac{1}{-i\omega C_g + [R_g - i\omega L_g]^{-1}} \tag{S8}$$

Assuming for the sake of simplicity that the dielectric function of metal is represented by the lossless Drude model and parametrised by the plasma frequency ω_p and ε_∞ we can obtain the resonant frequency from the poles of $\text{Im}\,(Z_{\text{total}})$. The solutions could proceed using the same algorithm as outlined in Ref.[4] However here we note that a dipolar plasmon frequency for our system, that assumes a dielectric spacer, can be obtained by scaling ε_∞ and ω_p^2 by $\frac{1-2d_\perp/R}{1+d_\perp/R}$. For the resonant plasmon wavelength λ_0 we obtain:

$$\lambda_0 = \lambda_p \sqrt{\varepsilon_\infty + 2\varepsilon_h(1 + 2\eta)\frac{1 + d_\perp/R}{1 - 2d_\perp/R}}, \tag{S9}$$

where λ_p is the plasma wavelength, $\lambda_p = 2\pi c/\omega_p$, and $\eta = C_g/C_{\text{fringe}}$ is the normalised gap capacitance.

S1.1.3 Gap capacitance

While Eq. S7 allows to estimate the role of nonlocality of the individual nanoparticles they clearly introduce corrections of the order of $d_\perp/R$ and therefore fail to incorporate the leading role of a small gap in the observable effect. To incorporate the latter we need to consider the change in gap capacitance induced by nonlocality. The simplest model of such kind would involve an adjustment of the gap thickness h by a nonlocal correction, resulting in the following modification of the normalized gap capacitance:

$$\eta = \frac{n_g^\chi}{\varepsilon_h} \ln\left(1 + \frac{R}{2(h - 2\alpha d_\perp)}\theta_{max}^2\right) \tag{S10}$$

Physically, this represents an additional contribution of $\alpha d_\perp E$ to the potential difference across the capacitor, that arises from potential discontinuities at the electrodes (for $\alpha = 1$). It is important to note that the in-plane gradient in Eq. S3 effectively violates the base assumption of the model, representing the field of each subdivision of curved electrode as equivalent to the field of a dihedral capacitor of the same size and orientation.[5] The unaccounted for contribution of the nearby points is what potentially makes the model to underestimate the nonlocality in a given setting. To compensate for this inconsistency we introduce a phenomenological parameter α which scales the contribution up to the observable values.

The value of χ, theoretically expected to be close to 2, was used in previous works[4] as a fine-tuning parameter to adjust the position of the NPoM resonance (in the local case, at $d_\perp = 0$), acting together with the size-dependent ε_∞. Here we follow the same recipe and obtain the unknown high-frequency dielectric constant $\varepsilon_\infty \approx 13.0$ by extrapolating the dataset[4] of a single-particle localised surface plasmon resonances in water (Figure S1). Subsequently, using the value $\chi = 2.02$ we obtain an excellent agreement of the wavelength of the fundamental NPoM resonance (805.6 nm) with that obtained in numerical simulations (804.9 nm). Moreover, estimating the thermal change of ε_∞ from our microscopic model

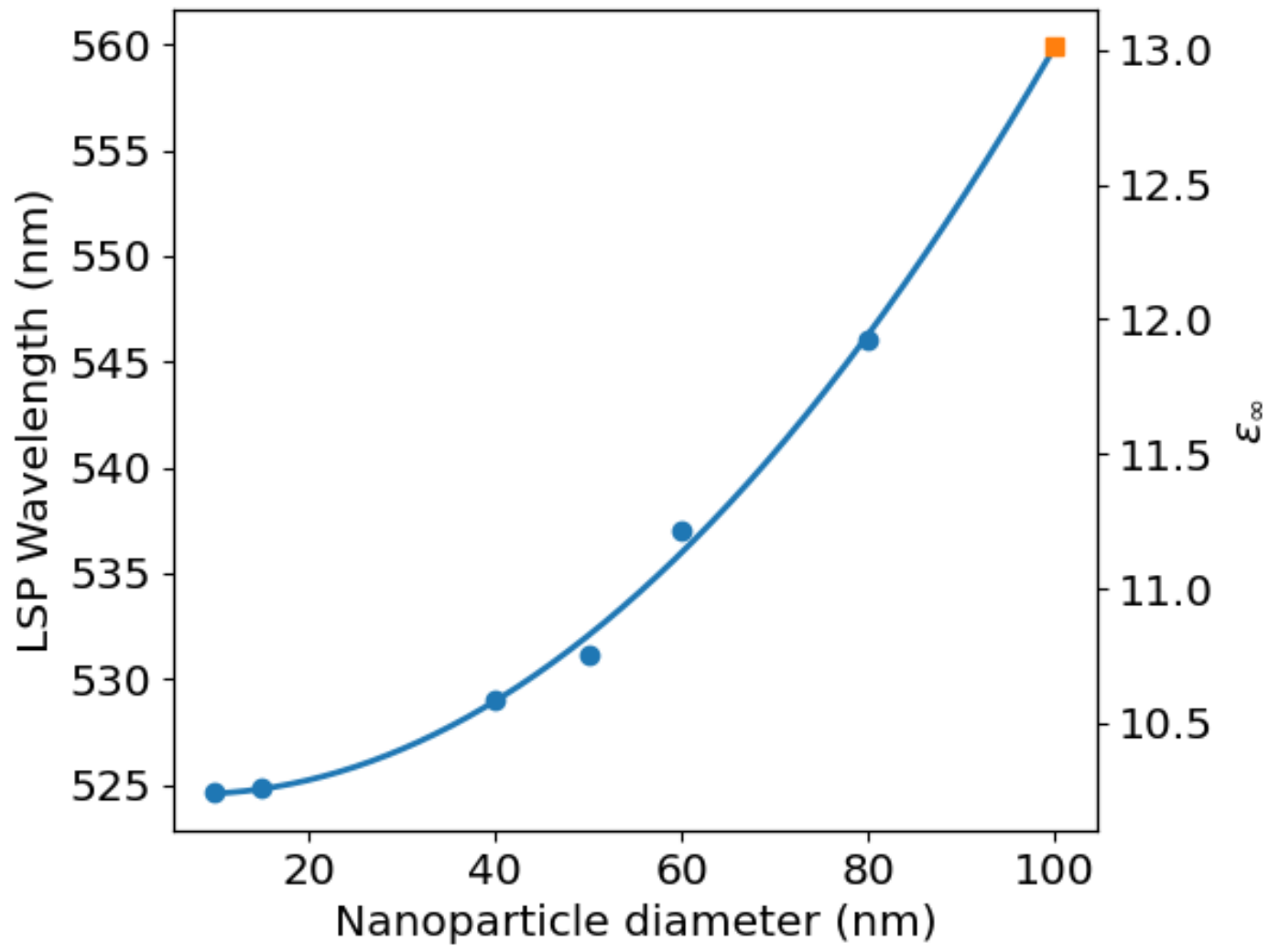

Figure S1: LSP resonances of spherical gold nanoparticles in water and recovered ε_∞. Data from Ref.[4] The solid line is a phenomenological polynomial extrapolation of the data. The orange square represents the nanoparticle diameter used in the present work (100 nm).

(Section S1.2) to be $\approx$ 0.22 upon heating to 2000 K, we are able to match the resonance positions at elevated electron temperatures without any extra free parameters.

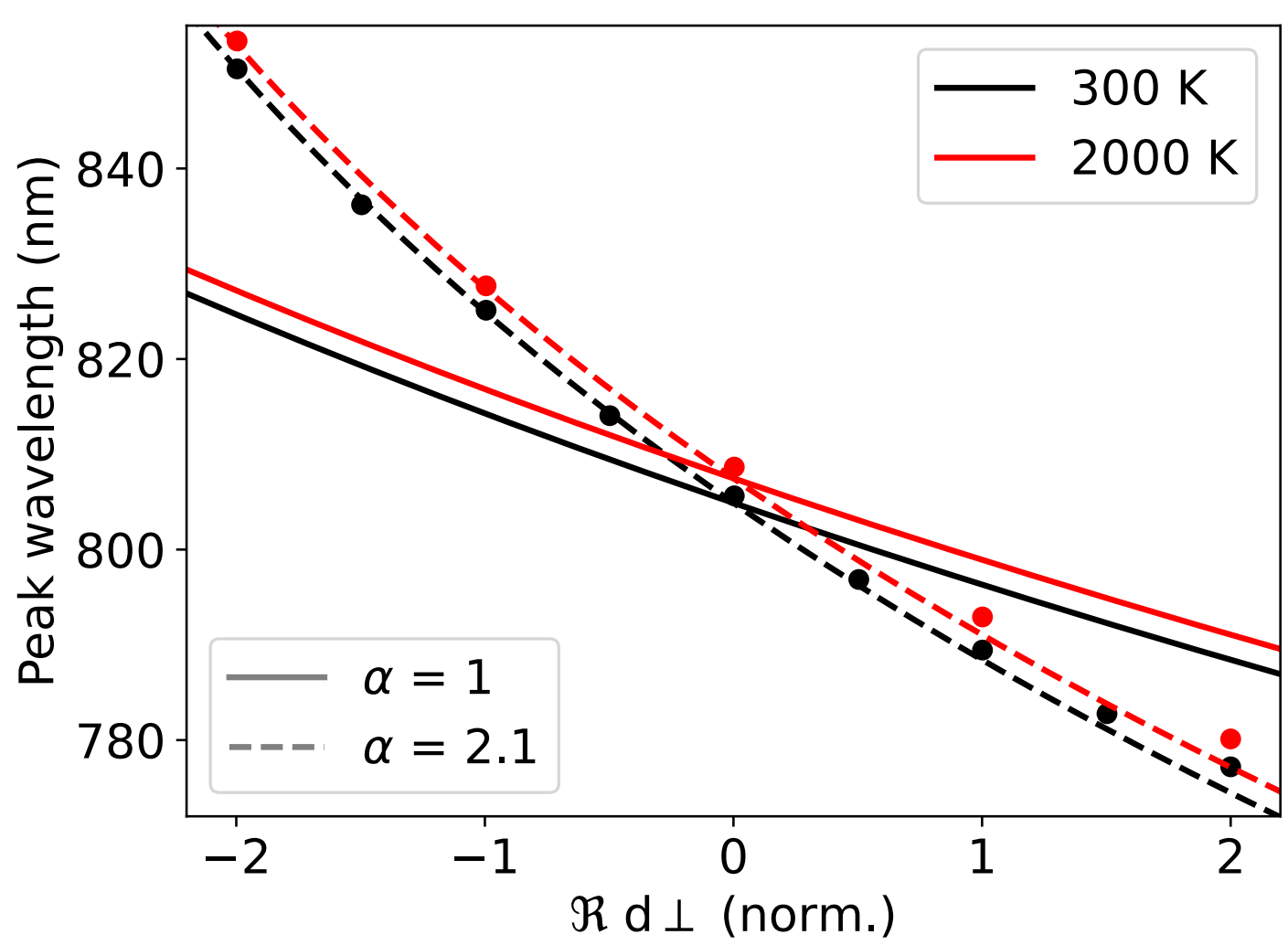

Figure S2: Positions of the NPoM resonance obtained from the nonlocal circuit model with $\alpha = 1$ (solid) and 2.1 (dashed), at the temperatures 300 K (black) and 2000 K (red). The points are the same as Fig. 4d in the main text.

S1.2 Time-depended DFT

TD-DFT calculations used in this study follow the route that is well established in the literature[6] and incorporate three main approximations: "jellium" treatment of the positive ionic background, adiabatic local density approximation (LDA) for the exchange-correlation potential and semi-classical approximation of the screening imposed by the *d*-electrons, known as the s-d model.[7,8] For simplicity and consistency with the established body of literature we also used Wigner interpolation for the exchange correlation energy. Although more accurate, newer expressions are available and frequently used in the literature[9] we did not notice a significant deviation of the results based on the exchange correlation approximation used.

The main deviation of our approach from the previous works is the use of microscopic temperature-dependent dielectric function of gold to devise the contribution of the *d*-band screening. The imaginary part of the interband dielectric function of gold, as stated in the main text, is given by:

$$\mathrm{Im}[\varepsilon_d(\omega, T_e)] = (\hbar\omega)^{-2}[A_X J_X(\omega, T_e) + A_{L_4^+} J_{L_4^+}(\omega, T_e) + A_{L_{5+6}^+} J_{L_{5+6}^+}(\omega, T_e)], \tag{S11}$$

where the joint densities of states J_i are calculated from the known band parameters through the energy distribution of the joint density of states:

$$J_i(\omega, t) = \int_{E_{min}}^{E_{max}} D_i(E, \omega) f(E, T_e, t) dE. \tag{S12}$$

Here f represents the Fermi-Dirac occupation factors, and the band parameters used to calculate D are given in Table S1. The weights A_i were chosen to match the room temperature dielectric function of gold (see Figure S6). The real part of ε was obtained by Kramers-Kronig transformation, and summed up with the Drude term, featuring additionally an ε_∞ which is also interpreted as a bound electron contribution and included in the calculation of the screening dielectric function, as discussed in the main text.

Table S1: Band-structure parameters near the X- and L-symmetry points used in the model for gold

Mass (m_0)										Energy (eV)				
$m^X_{p\parallel}$	$m^X_{p\perp}$	$m^X_{d\parallel}$	$m^X_{d\perp}$	$m^L_{p\parallel}$	$m^L_{p\perp}$	$m^{L_4^+}_{d\parallel}$	$m^{L_4^+}_{d\perp}$	$m^{L_{5+6}^+}_{d\parallel}$	$m^{L_{5+6}^+}_{d\perp}$	$\hbar\omega^X_7$	$\hbar\omega^X_6$	$\hbar\omega^L_{4+}$	$\hbar\omega^L_{4-}$	$\hbar\omega^L_{5+6}$
0.12	0.22	0.91	0.75	0.25	0.23	0.57	0.49	0.7	0.63	1.72	1.46	2.6	0.72	1.45

The groundstate and induced density calculations proceed as described elsewhere,[6,8,10,11] using $\varepsilon(0, Te)$ and $\varepsilon(\hbar\omega, T_e)$ in the screened Poisson equation and the long-wavelength approximation of external potential $\psi_{ext} = -2\pi z$. The first-order induced density δn is then given by:

$$\delta n = \int dz' \chi(z, z', \omega)[\psi_{ext}(z', \omega) + \psi_{ind}(z', \omega) + \delta V_{xc}(z', \omega)], \tag{S13}$$

where in adiabatic LDA $\delta V_{xc} = \frac{\delta V_{xc}[n(z,\omega)]}{\delta n(z,\omega)}$, and the linear response function χ is constructed from the ground state wave functions for the s-electrons.[10]

The dynamic force sum rule used to aid in the evaluation of $d_\perp$ in the presence of a polarisable medium has been extensively reported in the literature.[6,8] It is given by:

$$\begin{aligned} d_\perp &= \frac{\varepsilon - \varepsilon_d}{\varepsilon + 1} d_s + \frac{\varepsilon_d - 1}{\varepsilon + 1} d_d, \\ d_d &= \frac{\varepsilon_d - \varepsilon}{\varepsilon_d} d_s + \frac{\varepsilon + 1}{\varepsilon_d} \int_0^\infty z\,\delta n(z)\,dz, \\ d_s &= \frac{\varepsilon - \varepsilon_d}{\varepsilon} \left[\varepsilon_d \int_0^\infty \delta n(z)\,dz - (\varepsilon_d - 1) \left(\frac{\varepsilon_d}{\varepsilon + 1} \int_{-\infty}^0 f(z)\,dz + \int_0^\infty z\,\delta n(z)\,dz \right.\right. \\ &\qquad \left.\left. + \int_{-\infty}^0 f(z)\,dz \int_{-\infty}^z \delta n(z')\,dz' \right) \right]. \end{aligned} \tag{S14}$$

where $f(z) \equiv (n(z) - n_+(z))/n_0$, $n(z)$ is the groundstate density profile, and n_+ is a step-like density of the ionic background. Alternatively, as reported in previous works, this step can be bypassed by invoking a concept of nonlocal reflection coefficient.[9,11]

In addition, to demonstrate quantitative agreement of our screened calculations within their range of applicability, with the state-of-the-art approaches, we adapted the so-called

infinite barrier or specular reflection model (SRM) following Ref.[9] Within this formalism, the expression for the Feibelman parameter is given by:

$$d_{\mathrm{SRM}} = -\frac{2}{\pi}\frac{\varepsilon(\omega)}{\varepsilon(\omega)-1}\int_0^\infty \frac{1}{k^2}\left(\frac{1}{\varepsilon_L(\omega,k)} - \frac{1}{\varepsilon(\omega)}\right) dk, \tag{S15}$$

where $\epsilon_L(\omega, k)$ is an appropriate longitudinal dielectric function of metal. Choosing the hydrodynamics dielectric function $\varepsilon_L(\omega, k)$ with the parameter $\beta = \sqrt{\frac{3}{5}}v_F$ we can perform the integration analytically and obtain:

$$d_{\mathrm{SRM}} = i\frac{\varepsilon\beta}{\varepsilon-1}\frac{(\varepsilon_d/\varepsilon-1)^{\frac{3}{2}}}{\omega_p\sqrt{\varepsilon_d}}. \tag{S16}$$

This expression was used to produce the dashed curves in Figure 1b in the main text.

Lastly, to emphasize consistency of our choice of parameters, we note that for $r_s = 3$ one straightforwardly obtains $n = 5.96 \times 10^{28}$ m^{-3}, and thus $\omega_p = 9.09$ eV. One may set $r_s = 3.01$ to obtain an exact match to the value 9.02 eV up to a second digit, however, given that reported plasma frequency in gold varies based on sources in the literature between 8.5 and 9.1 eV (*e.g.* due to slight variations of the electron mass from the free electron mass, or variations of ionic potential at different crystal facets), we chose to set $r_s = 3$ for the sake of simplicity.

S2 Electromagnetic simulations

Numerical simulations were performed in COMSOL Multiphysics using the Electromagnetic Waves, Frequency Domain interface in the Wave Optics Module. We implement the mesoscopic boundary conditions in the COMSOL model through weak-form integrals within the scattering formulation of Maxwell's equations.[3,11]

$$\nabla \times \mu^{-1}\nabla \times E_{\mathrm{sc}} - \varepsilon(r,\omega)k^2 E_{\mathrm{sc}} = \Delta\varepsilon(r,\omega)k^2 E_{\mathrm{inc}} \tag{S17}$$

Here E_{inc} and E_{sc} are the incident and scattered fields while $\Delta\varepsilon$ is the permittivity contrast between the scattering object and its background. An induced in-plane surface current $K = -i\varepsilon_0\omega d_{||}[\varepsilon\nabla_{||}\varphi]$ and induced surface polarization $\pi = -\varepsilon_0 d_\perp[\nabla_\perp\varphi]\hat{n}$ can be expressed through the self-consistent potential φ ($E = -\nabla\varphi$, and $\hat{n}$ is an outward normal vector). To improve numerical efficiency and stability, $d_{||}$ was incorporated into the non-classical boundary conditions via a surface current term, while $d_\perp$ was treated using an auxiliary potential method (E^c_{sc} is a field with continuous parallel components[11]):

$$\begin{aligned} K(r) &= id_{||}\omega_{||}[D_{||}] \\ E_{\mathrm{sc}} &= E^c_{\mathrm{sc}} + \nabla\psi \end{aligned} \tag{S18}$$

The mesoscopic boundary conditions thus read:

$$[H_{||}] = K \times \hat{n} = i\omega d_{||}[D_{||}] \times \hat{n} \tag{S19}$$

$$[D_\perp] = (i\omega)^{-1}\nabla_{||} \cdot K = d_{||}\nabla_{||} \cdot [D_{||}] \tag{S20}$$

$$[E_{||}] = -\varepsilon_0^{-1}\nabla_{||}\pi = -d_\perp\nabla_{||}[E_\perp] \tag{S21}$$

$$[D_\perp] = 0 \tag{S22}$$

Further details can be found in Ref.[11] This approach is applicable to systems with moderate curvatures of all surfaces ($\kappa \ll |d_{\perp,||}|$) which holds true in the case of a large ($R = 50$ nm) nanoparticle considered here. We further omit higher-order contributions to the optical response (quadrupole etc.), electronic band quantization in ultrasmall objects (quantum size effects)[12] and quantum tunneling operative in plasmonic nanogaps below 1 nm.[13] In the computations, the two-dimensional model was expanded into the three-dimensional domain. The model geometry comprised a rectangular computational domain of a cubic shape with lateral dimensions of 500 nm, enclosed by a 150-nm-thick perfectly matched layer (PML) to ensure ideal absorption outside the active region of the system. The gold mirror layer with a thickness of 50 nm rests on an insulating ($\varepsilon = 2.25$) 200 nm-thick layer. In the calculations, we set $d_{||} = 0$ due to the charge neutrality and only consider variations of $d_{\perp}$.

S2.1 Consistency of the non-local computational model

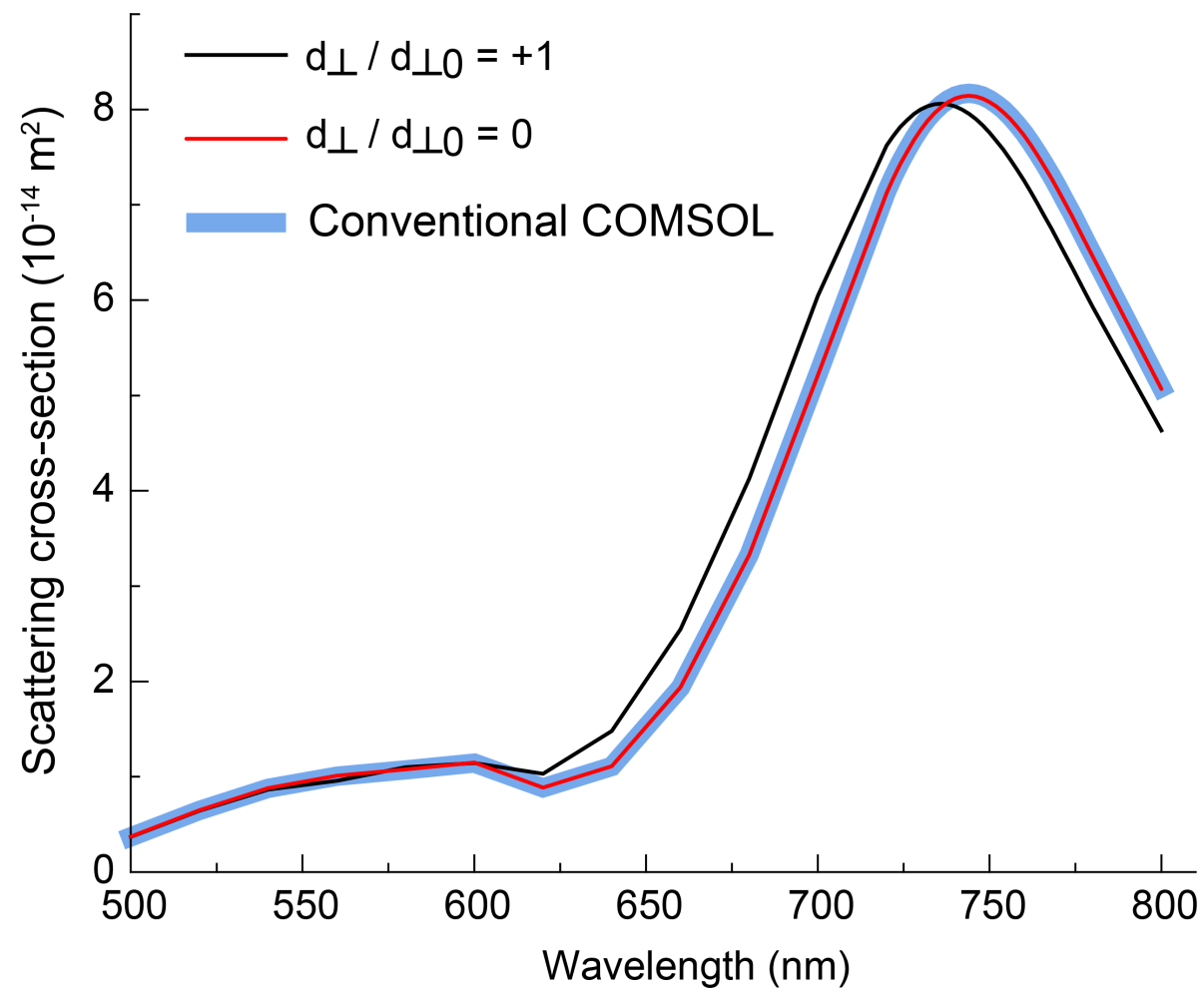

Figure S3: Scattering cross-section spectra of a single NPoM with $h = 2$ nm. The results of the non-local calculations with negligible non-locality $d_\perp = 0$ (red line) agree well with those of the conventional (local) calculations (thick blue line). The results obtained in a non-local model with $d_\perp = d_{\perp 0}$ are clearly different (black line).

S2.2 Second-order correction to the linear shift of the resonance

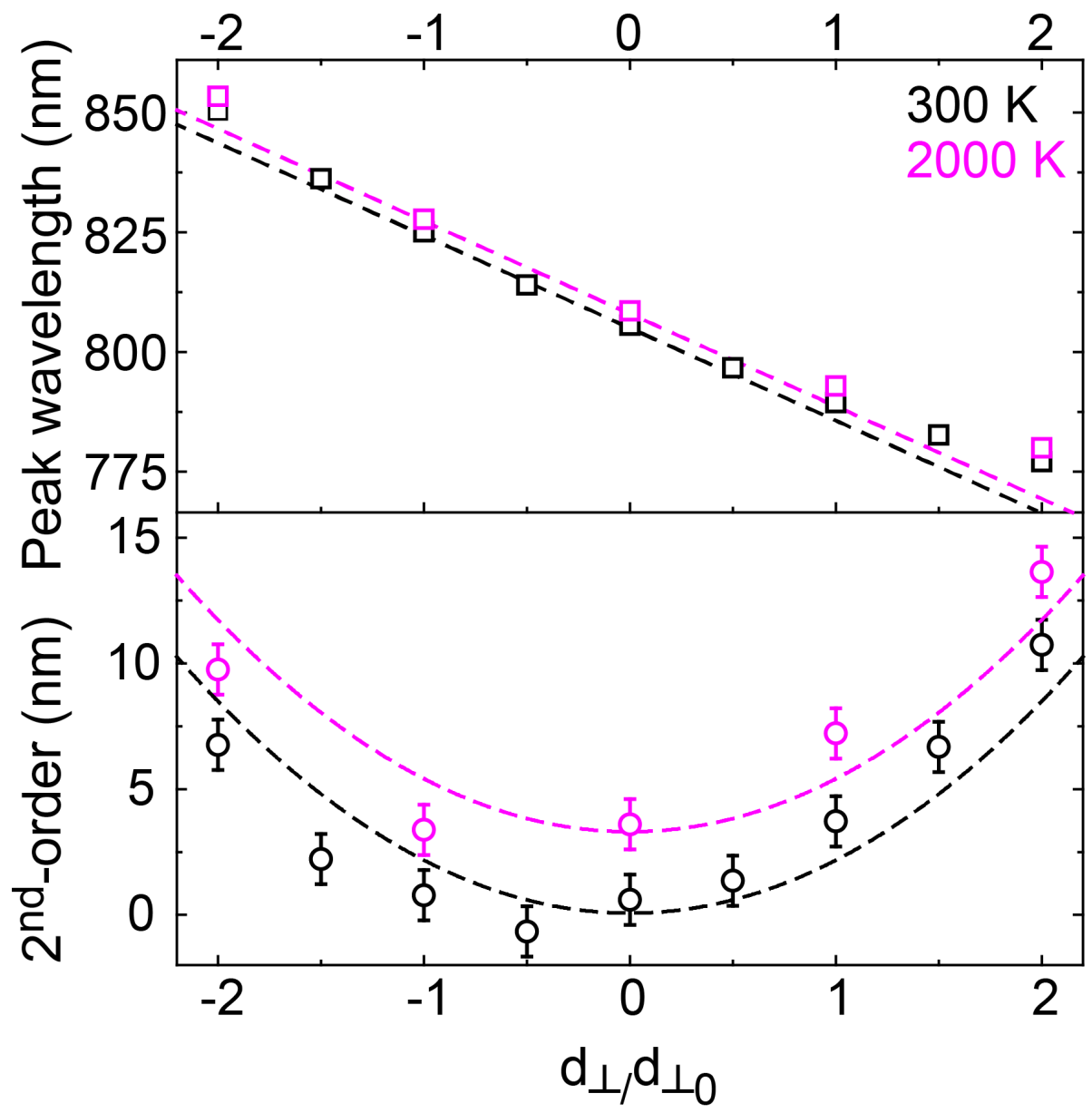

Figure S4: Detailed analysis of the role of non-locality in NPoM systems with cold (300 K) and laser-heated (2000 K) mirrors. Top panel: the dots show the peak positions extracted from the calculated spectra (cf. Fig. 4 in the main text), the parallel dashed lines are the linear fits with the shared slope. Bottom panel: the dots show the residuals (data from the top panel with linear fit subtracted) whereas the dashed lines illustrate the second-order correction to the linear approximation. The gap width $h = 1$ nm.

S2.3 Non-equilibrium NPoM systems: symmetry role

The built-in asymmetry between the mirror and the particle in NPoM reportedly results in interesting emission properties.[14] In our approach, the asymmetry is further emphasized by the hot electron injection into the mirror and thus its elevated T_e. To verify the anticipated degree of asymmetry, we flip the electron temperatures and numerically compare a system depicted in Fig. 2 to its opposite, that is, where $T_e = 2000$ K on the particle while the mirror remains cold. As a measure for the asymmetry, we compare it with the unexcited NPoM where both the mirror and the particle are kept at $T_e = 300$ K. The results (Fig. S5) indicate that the symmetric contribution to scattering dominates although the resonance shift is slightly stronger when the mirror is hot. This effect should vanish for larger nanoparticles, approaching the symmetric waveguide geometry whereas for even smaller ones a more pronounced asymmetric response can be expected.

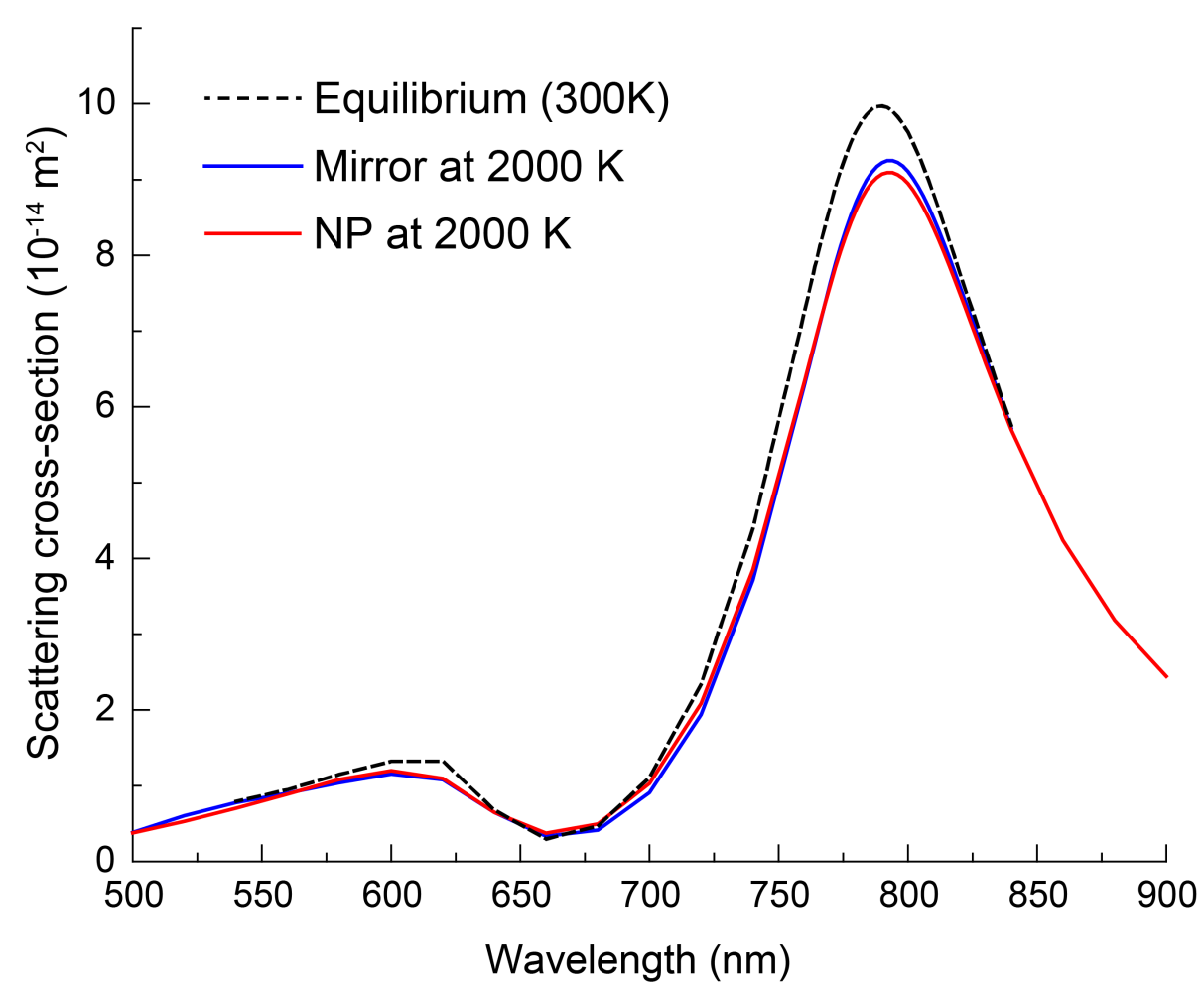

Figure S5: Calculated scattering cross-section spectra of a NPoM with $h = 1$ nm when both nanoparticle and mirror are in the equilibrium at 300 K (black dashed line), as well as when the nanoparticle (red) or the mirror (blue) is laser-heated up to 2000 K.

S2.4 Dielectric function of Au at elevated temperatures

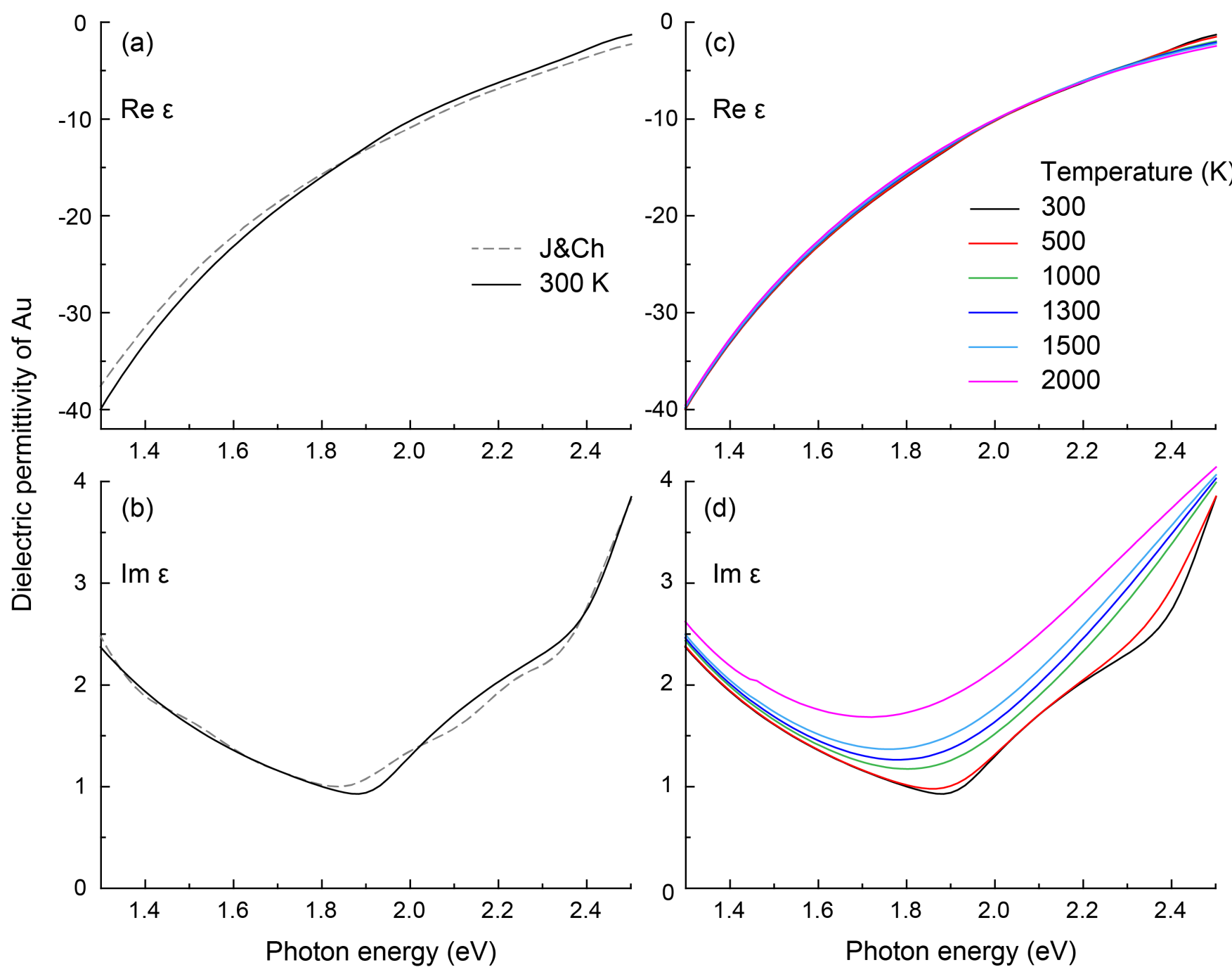

Figure S6: Calculated dielectric function of Au using the approach from Ref.[15] In panels (a-b), the results of our calculations for the real (a) and imaginary (b) parts are compared against the data from Johnson and Christy[16] (dashed lines). In panels (c-d), the evolution of the calculated dielectric function with temperature is shown.

S2.5 Linewidths and Q-factors in NPoM systems with non-locality

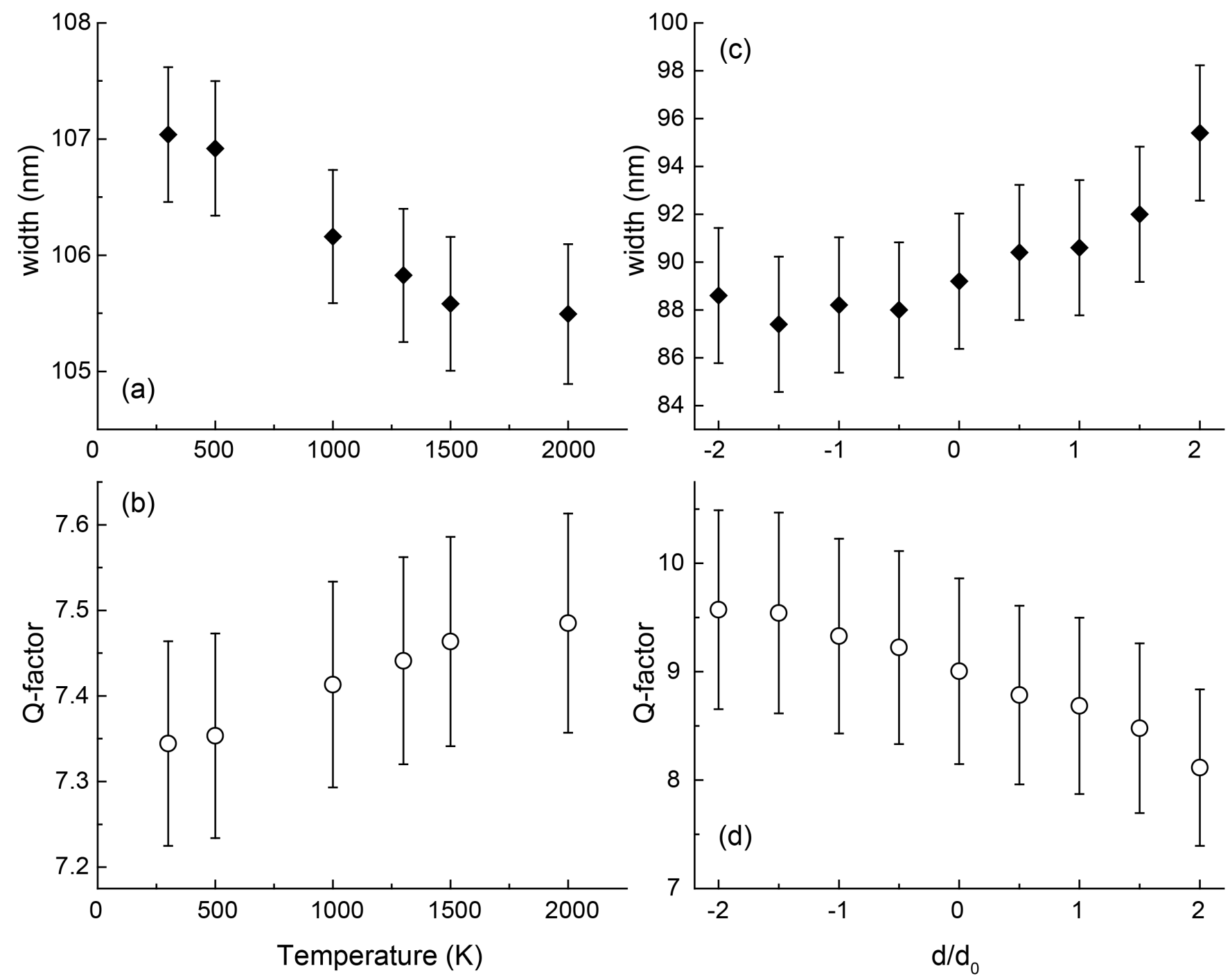

Figure S7: Linewidths (a,c) and recalculated Q-factors of the plasmon gap mode in non-local non-equilibrium NPoM systems. The linewidths are obtained from fitting the calculated scattering cross-section spectra with a Lorentzian in the vicinity of the resonance. The Q-factors are calculated as a ratio of the resonance frequency to the linewidth in the frequency domain, $Q = \omega_0/\Delta\omega$. As in the main text, in (a-b) the horizontal axis refers to the electronic temperature in the mirror whereas the nanoparticle remains at room temperature. In (c-d), the Feibelman non-locality $d_\perp$ is varied with respect to the d_0 values calculated in Ref.[9]

References

(1) Engheta, N.; Salandrino, A.; Alù, A. Circuit Elements at Optical Frequencies: Nanoinductors, Nanocapacitors, and Nanoresistors. *Phys. Rev. Lett.* **2005**, *95*, 095504.

(2) Gonçalves, P. A. D.; Christensen, T.; Rivera, N.; Jauho, A.-P.; Mortensen, N. A.; Soljačić, M. Plasmon–emitter interactions at the nanoscale. *Nature Communications* **2020**, *11*, 366.

(3) Christensen, T.; Yan, W.; Jauho, A.-P.; Soljačić, M.; Mortensen, N. A. Quantum Corrections in Nanoplasmonics: Shape, Scale, and Material. *Phys. Rev. Lett.* **2017**, *118*, 157402.

(4) Benz, F.; de Nijs, B.; Tserkezis, C.; Chikkaraddy, R.; Sigle, D. O.; Pukenas, L.; Evans, S. D.; Aizpurua, J.; Baumberg, J. J. Generalized circuit model for coupled plasmonic systems. *Opt. Express* **2015**, *23*, 33255–33269.

(5) Hudlet, S.; Saint Jean, M.; Guthmann, C.; Berger, J. Evaluation of the capacitive force between an atomic force microscopy tip and a metallic surface. *The European Physical Journal B - Condensed Matter and Complex Systems* **1998**, *2*, 5–10.

(6) Liebsch, A. *Electronic Excitations at Metal Surfaces*; Physics of Solids and Liquids; Springer New York, 1997.

(7) Liebsch, A. Surface plasmon dispersion of Ag. *Phys. Rev. Lett.* **1993**, *71*, 145–148.

(8) Liebsch, A.; Schaich, W. L. Influence of a polarizable medium on the nonlocal optical response of a metal surface. *Phys. Rev. B* **1995**, *52*, 14219–14234.

(9) Echarri, A. R.; Gonçalves, P. A. D.; Tserkezis, C.; de Abajo, F. J. G.; Mortensen, N. A.; Cox, J. D. Optical response of noble metal nanostructures: quantum surface effects in crystallographic facets. *Optica* **2021**, *8*, 710–721.

(10) Liebsch, A. Dynamical screening at simple-metal surfaces. *Phys. Rev. B* **1987**, *36*, 7378–7388.

(11) Yang, Y.; Zhu, D.; Yan, W.; Agarwal, A.; Zheng, M.; Joannopoulos, J. D.; Lalanne, P.; Christensen, T.; Berggren, K. K.; Soljačić, M. A general theoretical and experimental framework for nanoscale electromagnetism. *Nature* **2019**, *576*, 248–252.

(12) Halperin, W. P. Quantum size effects in metal particles. *Rev. Mod. Phys.* **1986**, *58*, 533–606.

(13) Esteban, R.; Borisov, A. G.; Nordlander, P.; Aizpurua, J. Bridging quantum and classical plasmonics with a quantum-corrected model. *Nature Communications* **2012**, *3*, 825.

(14) Li, G.-C.; Lei, D.; Qiu, M.; Jin, W.; Lan, S.; Zayats, A. V. Light-induced symmetry breaking for enhancing second-harmonic generation from an ultrathin plasmonic nanocavity. *Nature Communications* **2021**, *12*, 4326.

(15) Guerrisi, M.; Rosei, R.; Winsemius, P. Splitting of the interband absorption edge in Au. *Phys. Rev. B* **1975**, *12*, 557–563.

(16) Johnson, P. B.; Christy, R. W. Optical Constants of the Noble Metals. *Phys. Rev. B* **1972**, *6*, 4370–4379.
